# External validation of the CARDOT score for predicting respiratory complications after thoracic surgery

**DOI:** 10.1186/s12871-024-02685-5

**Published:** 2024-08-30

**Authors:** Tanyong Pipanmekaporn, Pakaros Kitswat, Prangmalee Leurcharusmee, Thanaporn Runraksar, Nutchanart Bunchungmongkol, Jiraporn Khorana, Apichat Tantraworasin, Panuwat Lapisatepun, Surasak Saokaew

**Affiliations:** 1 Department of Anesthesiology, Faculty of Medicine, Chiang Mai University, Intavarorote Rd, Muang Chiang Mai District, Chiang Mai, 50200 Thailand; 2 Department of Biomedical informatics and Clinical Epidemiology, Faculty of Medicine, Chiang Mai University, Chiang Mai, 50200 Thailand; 3 Department of Surgery, Faculty of Medicine, Chiang Mai University, Chiang Mai, 50200 Thailand; 4 Division of Social and Administrative Pharmacy (SAP), Department of Pharmaceutical Care, School of Pharmaceutical Sciences, University of Phayao, Phayao, 56000 Thailand; 5 Center of Excellence in Bioactive Resources for Innovative Clinical Applications, Chulalongkorn University, Bangkok, 10330 Thailand; 6 Unit of Excellence on Clinical Outcomes Research and IntegratioN (UNICORN), School of Pharmaceutical Sciences, University of Phayao, Phayao, 56000 Thailand; 7 Department of Anesthesiology, Sunpasitthiprasong Hospital, Ubon Ratchathani, 34000 Thailand; 8 PRINC Lumphun Hospital, Lumphun, 51000 Thailand

**Keywords:** Postoperative respiratory complication, Postoperative pulmonary complication (PPC), Thoracic surgery, Risk score, Neutrophil-lymphocyte ratio

## Abstract

**Background:**

The CARDOT scores have been developed for prediction of respiratory complications after thoracic surgery. This study aimed to externally validate the CARDOT score and assess the predictive value of preoperative neutrophil-to-lymphocyte ratio (NLR) for postoperative respiratory complication.

**Methods:**

A retrospective cohort study of consecutive thoracic surgical patients at a single tertiary hospital in northern Thailand was conducted. The development and validation datasets were collected between 2006 and 2012 and from 2015 to 2021, respectively. Six prespecified predictive factors were identified, and formed a predictive score, the CARDOT score (chronic obstructive pulmonary disease, American Society of Anesthesiologists physical status, right-sided operation, duration of surgery, preoperative oxygen saturation on room air, thoracotomy), was calculated. The performance of the CARDOT score was evaluated in terms of discrimination by using the area under the receiver operating characteristic (AuROC) curve and calibration.

**Results:**

There were 1086 and 1645 patients included in the development and validation datasets. The incidence of respiratory complications was 15.7% (171 of 1086) and 22.5% (370 of 1645) in the development and validation datasets, respectively. The CARDOT score had good discriminative ability for both the development and validation datasets (AuROC 0.789 (95% CI 0.753–0.827) and 0.758 (95% CI 0.730–0.787), respectively). The CARDOT score showed good calibration in both datasets. A high NLR (≥ 4.5) significantly increased the risk of respiratory complications after thoracic surgery (*P* < 0.001). The AuROC curve of the validation cohort increased to 0.775 (95% CI 0.750–0.800) when the score was combined with a high NLR. The AuROC of the CARDOT score with the NLR showed significantly greater discrimination power than that of the CARDOT score alone (*P* = 0.008).

**Conclusions:**

The CARDOT score showed a good discriminative performance in the external validation dataset. An addition of a high NLR significantly increases the predictive performance of CARDOT score. The utility of this score is valuable in settings with limited access to preoperative pulmonary function testing.

**Supplementary Information:**

The online version contains supplementary material available at 10.1186/s12871-024-02685-5.

## Introduction

Respiratory complications are among the most common complications after thoracic surgery and often become a leading cause of perioperative morbidity and mortality, prolonged length of hospital stays, and increased medical cost [1–3]. The incidence of respiratory complications after thoracic surgery varies between 12 and 30% depending on its definition among studies, study populations, and study designs [2, 4, 5]. The spectrum of respiratory complications generally ranges from atelectasis and pneumonia to the most severe form of adult respiratory distress syndrome [6]. Empyema thoracis, chylothorax, and bronchopleural fistula is classified as postoperative pulmonary complications in accordance with the European Perioperative Clinical Outcomes definitions [7].

Numerous risk scores have been developed to predict postoperative pulmonary complications (PPCs) in general surgery [8]. While some clinical risk scores, like Assess Respiratory Risk in Surgical Patients in Catalonia (ARISCAT) [9], the Score for prediction of postoperative pulmonary complication (SPORC) [10], a clinical risk score named the surgical lung injury prediction (SLIP) [11, 12], the Local Assessment of Ventilatory Management During General Anesthesia for Surgery (LAS VEGAS) [13], and the Clinical Prediction Rule for Pulmonary Complications (CPRPC) [2, 4], have shown utility in specific settings, their applicability to thoracic surgery is limited due to the distinct physiological challenges of these patient population. In 2015, the CARDOT score, developed to predict respiratory complications after thoracic surgery, incorporates chronic obstructive pulmonary disease (COPD), American Society of Anesthesiologists physical status (ASA PS), right-sided operation, operative duration, and preoperative oxygen saturation on room air, and thoracotomy [14]. This score demonstrated good discriminative performance in development and internal validation with the areas under the receiver operating characteristic curves (AuROC) of 0.789 and 0.758, respectively [14]. However, external validation and further research are required to confirm its generalizability. To assess the potential impact of temporal trends on the performance of CARDOT score, patient characteristics, anesthesia techniques, and surgical practices are compared between development and validation cohorts.

Neutrophil-to-lymphocyte ratio (NLR) is a well-established marker of systemic inflammation and disease severity, easily obtained from routine complete blood counts. Normal NLR values range from 1 to 2 in healthy adults, with elevations (> 3) indicating a pathological state [15–17]. Cutoff values for NLR vary across diseases, and a higher NLR is consistently linked to adverse outcomes in conditions such as COPD [18], interstitial lung disease [18], lung cancer [19, 20], and COVID-19 [21]. As NLR data were unavailable for the development set (year 2006–2012), it was not incorporated into the CARDOT score. Its inclusion was limited to the validation set. This study represents the initial exploration of NLR as a potential predictor of PPCs following thoracic surgery. This study aimed to externally validate the CARDOT score and assess the incremental predictive value of preoperative NLR for PPCs.

## Methods

This validation study was performed under transparent reporting of a multivariable prediction model for individual prognosis or diagnosis (TRIPOD) reporting guideline and the checklist [22] is provided in Supplementary Table S1.

### Source of data

This retrospective study (validation cohort) was performed in a cohort of consecutive adult patients who underwent noncardiac thoracic surgery at Chiang Mai University Hospital between January 2015 and December 2021. The study was approved by the Ethics Committee of Chiang Mai University (CMU) Hospital- STUDY CODE: ANE-2565-08986/Research ID 8986. Patient informed consent was waived due to the retrospective study design. The data from the development cohort were retrospectively collected at the same institution between 1 January 2006 and 31 December 2012.

### Participants

The inclusion criteria were adult patients aged 18 years or older who underwent noncardiac thoracic surgery. The exclusion criteria included preoperative endotracheal tube intubation, surgical procedures related to thoracic injury, cardiac and orthopedic surgery, death during the intraoperative period and missing patient demographic information (e.g. age, gender, and ASA PS).

### Predictors

The electronic medical records were extensively and independently reviewed by two investigators (PK, TR). Patient characteristics including age, gender, weight, height, body mass index (BMI), ASA PS, smoking status, comorbidities, preoperative oxygen saturation measured by pulse oximetry (SpO2) breathing room air, preoperative chemotherapy, respiratory infection within one month and laboratory investigation were recorded. COPD was defined as one or more of the following conditions: functional disability, hospitalization prior to treatment for COPD, requiring treatment with bronchodilators, and forced expiratory volume in one second (FEV1) < 75% of the predicted value [6]. Surgical details consisted of the side of the operation, type of surgery (elective or emergency surgery), operative approach (thoracotomy or video-assisted thoracoscopic surgery (VATS)), surgical procedures (explorative thoracotomy, wedge resection, segmental resection, lobectomy, bilobectomy, or pneumonectomy) and duration of surgery. Anesthetic details included anesthetic techniques, amount of perioperative fluid administered, type of analgesia, duration of anesthesia, blood or blood product administration and respiratory events during the intraoperative period. Preoperative laboratory parameters including complete blood count, NLR, serum creatinine, albumin, and ppoFEV1% were measured. The NLR was defined as the absolute neutrophil count divided by the absolute lymphocyte count [16]. Pulmonary function test (PFT) data, including forced expiratory volume in one second (FEV1) and ppoFEV1%, were collected for a subset of patients in both cohorts.

The standard techniques of general anesthesia were performed for all thoracic patients at our institute, General anesthesia was induced by intravenous anesthetic drugs with propofol, and cis-atracurium or rocuronium was administered as a neuromuscular blocking agent. Anesthesia was maintained using a volatile anesthetic, which consisted of sevoflurane or desflurane, and intermittent boluses of intravenous fentanyl. A double-lumen endotracheal tube (DLT), bronchial blocker or endotracheal tube was appropriately selected for the lung separation method. A fiberoptic bronchoscope was routinely used for all patients who received DLTs or bronchial blockers. During one lung ventilation, a fraction of inspired oxygen of 1.0 and volume-controlled ventilation were used with a tidal volume of 6–8 ml/kg, maintaining a peak inspiratory pressure < 30 cmH20. The fraction of inspired oxygen was reduced and adjusted to maintain SpO2 > 94%. The respiratory rate was adjusted to maintain end-tidal carbon dioxide < 45 mmHg. Postoperative analgesia consisted of intravenous opioid infusion or thoracic epidural or thoracic paravertebral nerve block depending on the attending anesthesiologists.

### Outcomes

The primary outcome of this study was respiratory complications which were included as composite outcomes within 30 days after the operation. A patient was considered to have respiratory complications if he or she developed at least one of the following respiratory events. The presence of respiratory complications was determined by reviewing medical records of at least one of the following respiratory events: bronchospasm, desaturation, atelectasis, upper airway obstruction, pneumonia, respiratory failure, or acute respiratory distress syndrome (ARDS) [9, 23]. Bronchospasm was defined as newly detected expiratory wheezing or evidence of bronchodilator treatment. Desaturation was defined as a decrease in SpO2 to less than 90% in three minutes or a decrease in SpO2 to less than 85% at that time and the need for oxygen therapy. Atelectasis was indicated by lung opacification with a mediastinum shift, a hilum, or hemidiaphragm shift toward the affected area on chest radiograph and the need for bronchoscopy. Upper airway obstruction was defined as a newly detected stridor. Pneumonia was defined as fever with new lung infiltration on chest radiograph or purulent tracheal secretion confirmed by Gram strain or sputum culture and requiring antibiotics. Respiratory failure was defined as postoperative ventilator dependence for more than 24 h or reintubation for controlled ventilation. ARDS was defined as acute onset with a PaO2/FIO2 ≤ 200 mmHg and bilateral infiltration was observed on chest radiography, with no evidence of left atrial hypertension.

### Sample size

To ensure the precise estimation of key parameters in the prediction model, the sample size estimation for external validation of the prediction model for a binary outcome was calculated using the specific criteria proposed by Riley et al. in 2020 [24]. Based on the development study, the AuROC was 0.789. The incidence of respiratory complications was 15.8% (0.158), with a significance level (α) of 0.05 and a power (β) of 0.80 [14]. The required sample size was at least 366 when six candidate predictor parameters (CARDOT prediction score) were employed to achieve sufficient statistical power for external validation.

### Missing data

During development, some variables with missing data (< 5% missing) e.g. BMI, and preoperative hemoglobin were replaced using the observed frequency method by median imputation. In this study, patients with missing key data variables (e.g., ASA PS, SpO2 on room air, COPD status, surgical details, postoperative respiratory complications) were excluded to ensure a complete case analysis.

### Statistical analysis

All the statistical analyses were performed using Stata version 16.0 software (StataCorp LP, College Station, Texas, USA). Comparisons between the developmental and validation datasets were carried out. The descriptive data are presented as numbers and percentages for categorical variables. Continuous variables are expressed as the mean and standard deviation (SD) or median and 25th and 75th percentiles for continuous data depending on their distribution. Univariable analysis was carried out using an exact probability test for categorical variables and an unpaired t-test or Mann–Whitney U test for continuous variables. The multivariable analysis was performed using a logistic regression model with six predefined predictors from the developmental model. A two-tailed analysis with *P* < 0.05 was considered to indicate statistical significance.

CARDOT scores ranging from 0 to 13 points were assigned to each predictor (Table 1). In the development cohort, patients with a total score of 0 to 5.5 points were classified into the low-risk group for the occurrence of respiratory complications, and patients with a total score of 6 to 7.5 points and a score > 7.5 points were classified into the moderate risk and high risk groups for respiratory complications, respectively. This classification of groups was repeated with adapted threshold levels (< 6 and ≥ 6), which were recalculated by grouping the patients into tertiles based on the raw CARDOT score values of the validation dataset. The sensitivity, specificity, positive predictive value (PPV), negative predictive value (NPV), and the positive and negative likelihood ratio tests of both the original and the adapted threshold levels were calculated.

**Table 1:** Six predictors of CARDOT score with score assignment

Predictors	Score
COPD	2
ASA PS ≥ 3	3.5
Right -sided operation	1.5
Duration of surgery > 180 min	2
Preoperative SpO2 at room air	
91–95%	1
≤90%	2.5
Thoracotomy	1.5

*COPD* Chronic obstructive pulmonary disease, *ASA PS* American Society of Anesthesiologists physical status, *SpO2* oxygen saturation measured by pulse oximetry

The validation dataset was compared with the development dataset by the AuROC. A comparison of the probability of respiratory complications is shown in the bar chart with an error bar. The calibration curves were generated to graphically compare the predictive ability of the scoring system in both datasets. The Hosmer–Lemeshow test was used to assess the goodness-of-fit statistics comparing the agreement between the observed and expected score values are presented.

## Results

**Fig. 1:**
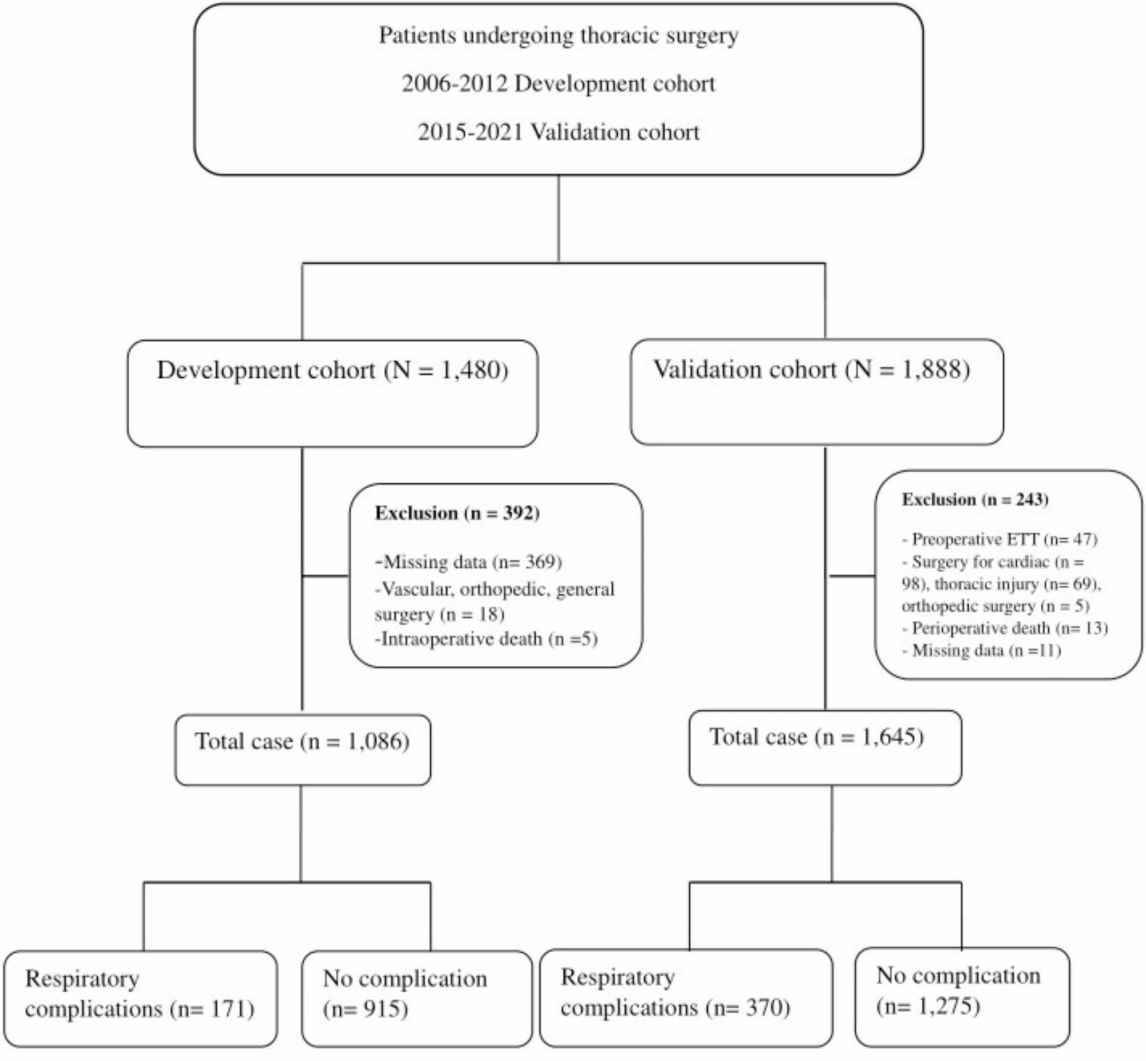
Study flow diagram

In the development dataset, a total of 1086 patients were recruited. One thousand, six hundred forty-five patients were included in the validation dataset. The incidence of respiratory complications in the validation dataset was significantly greater than that in the development dataset (15.7% (171 of 1086) vs. 22.5% (370 of 1645), *P* < 0.001). The recruitment flow chart is presented in Fig. 1.

The comparative baseline characteristics between the development and validation cohorts are shown in Table 2. The median values of ppoFEV1 in the development dataset (56 (46.5–60)) and validation dataset (69.2 (57-81.5)) were described.

**Table 2:** Patient characteristics, surgical and anesthetic details of the development and validation datasets

Patient characteristics	Development dataset (*n* = 1086)	Validation dataset (*n* = 1645)	*P*
Age (year)	53.4 ± 15.4	57.3 ± 14.6	< 0.001
Male, *n* (%)	654 (60.2)	883 (53.7)	0.001
ASA PS, *n* (%)			0.542
I	138 (12.7)	186 (11.3)	
II	678 (62.4)	1,036 (63.1)	
III	270 (24.9)	420 (25.6)	
BMI (kg/m^2^)	21.1 ± 3.7	22.0 ± 4.0	< 0.001
Co-morbidity, *n* (%)			
Hypertension	277 (25.5)	592 (36.0)	< 0.001
Diabetes Mellitus	105 (9.7)	226 (13.7)	0.001
COPD	117 (10.8)	118 (7.2)	0.001
Renal disease	121 (11.1)	129 (7.8)	0.008
Coronary artery disease	20 (1.9)	54 (3.3)	0.010
Current smoker, *n* (%)	532 (49.1)	621 (37.8)	< 0.001
Preoperative SpO2 at room air, *n* (%)			0.001
≥ 96%	807 (77)	1,306 (79.4)	
91-95%	246 (23)	280 (17.0)	
≤ 90%	33 (3)	59 (3.6)	
Preoperative hemoglobin (g/dl)	12.1 ± 1.8	12.0 ± 3.7	0.663
Preoperative albumin (g/dl)	3.5 (2.8-4)	4 (3.4–4.4)	< 0.001
Surgical details Diagnosis, *n* (%)			< 0.001
Benign lesion	128 (12)	144 (8.8)	
Infection	298 (27.7)	358 (21.8)	
Malignancy	573 (53.2)	976 (59.4)	
Disease of esophagus	4 (0.4)	1 (0.10)	
Tracheal lesion	1 (0.1)	6 (0.4)	
Other	82 (6.8)	160 (9.6)	
Emergency operation, *n* (%)	289 (27.0)	465 (28.3)	0.445
Right-sided operation, *n* (%)	690 (63.5)	916 (55.7)	< 0.001
Surgery on both sides, *n* (%)	10 (0.9)	94 (5.7)	
Thoracotomy, *n* (%)	822(75.6)	388 (23.5)	< 0.001
Type of surgical procedures, *n* (%)			< 0.001
Explorative thoracotomy	573 (52.8)	873 (53.1)	
Wedge resection / segmentectomy	146 (13.4)	370 (22.5)	
Lobectomy	344 (31.7)	389 (23.6)	
Pneumonectomy	23 (2.1)	13 (0.8)	
Duration of surgery (min)	135 (95–200)	120 (75–180)	0.004
Anesthetic profiles			
Fluid intake (ml)	1100 (680–1650)	750 (480–1150)	
Blood transfusion (ml)	200 (0-310)	0 (0–0)	< 0.001
Duration of anesthesia (min)	145 (105–190)	165 (120–210)	< 0.001
Length of hospital stay (days)	7 (5–10)	7 (5–12)	0.495

Data are presented as the mean ± standard deviation, median and 25th and 75th percentiles and the number of patients (%). *COPD* Chronic obstructive pulmonary disease, *ASA PS* American Society of Anesthesiologist physical status, *SpO2* oxygen saturation measured by pulse oximetry

The data are presented as the mean ± standard deviation, median and 25th and 75th percentiles and the number of patients (%). *COPD* Chronic obstructive pulmonary disease, *ASA PS* American Society of Anesthesiologists physical status, *SpO2* oxygen saturation measured by pulse oximetry.

**Fig. 2:**
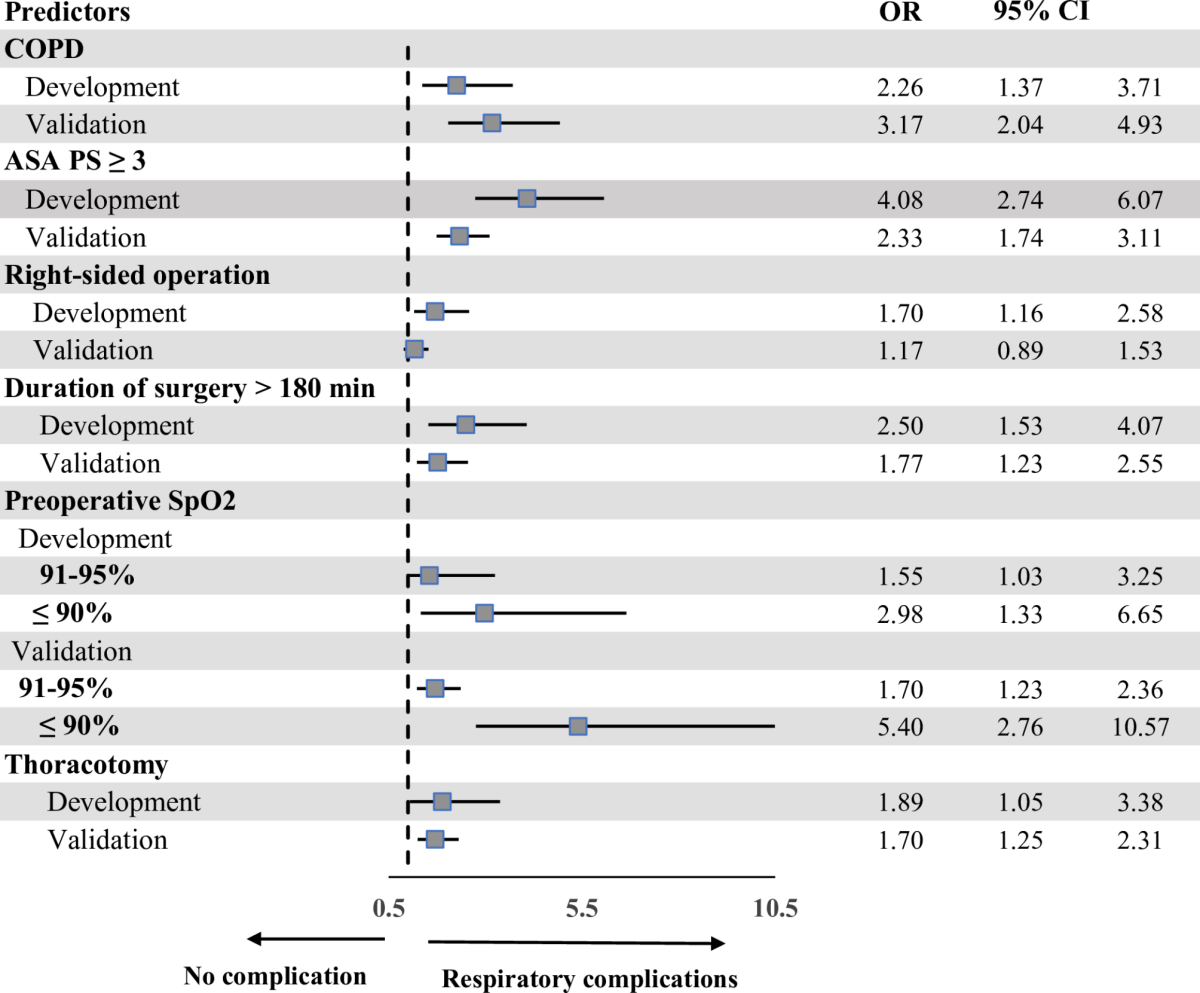
Risk ratio plots of predictors of the CARDOT score in the development and validation datasets

**Fig. 3:**
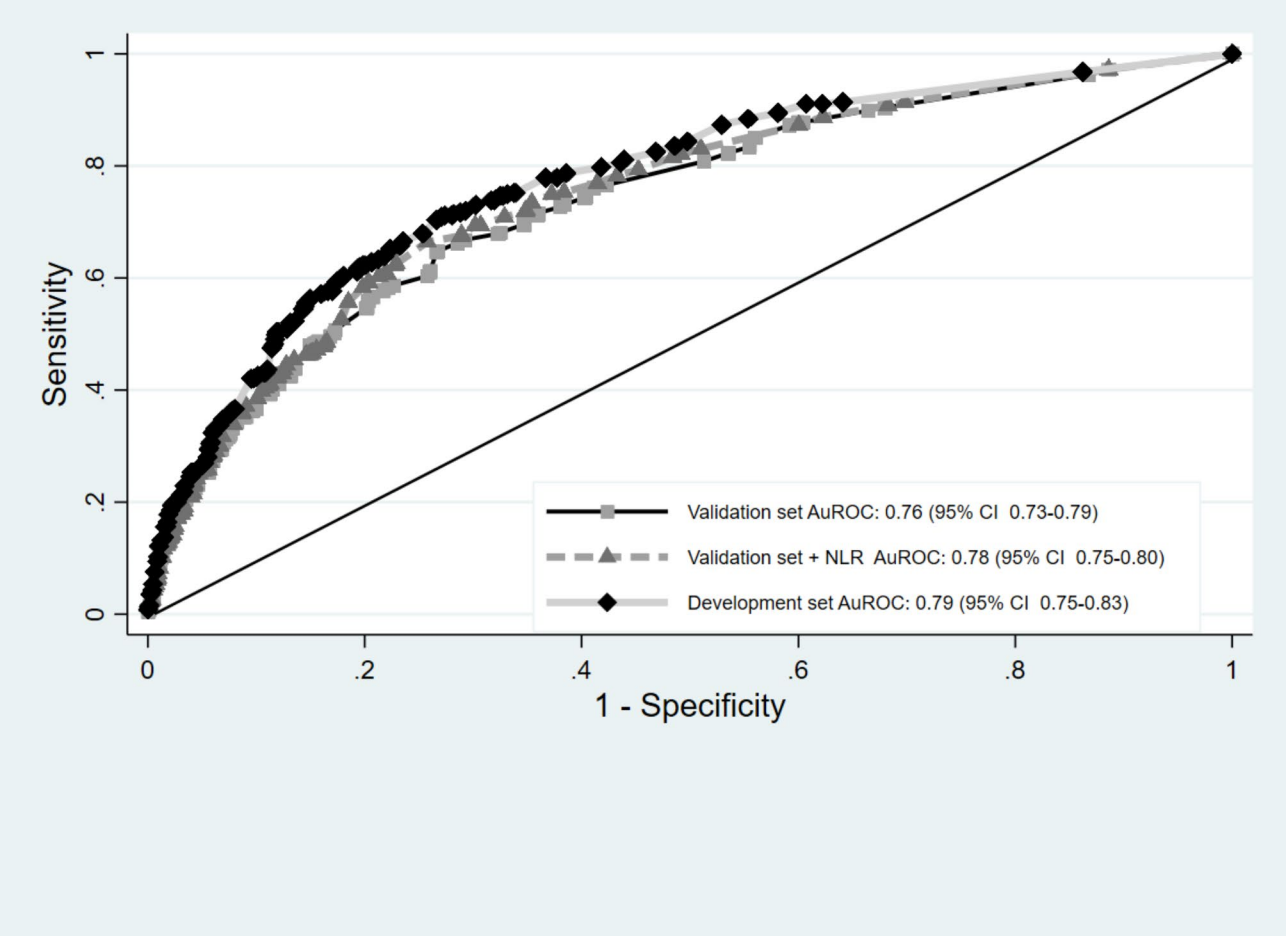
Comparative the area under the receiver operating characteristic of the development dataset, validation dataset with or without neutrophil-to-lymphocyte ratio (NLR). Development dataset (solid line with square marker), validation dataset (solid line with diamond marker), and validation dataset with NLR (dash line with triangle marker) predicted by risk scoring scheme (curve lines) and a 50% chance prediction (diagonal line)

**Fig. 4:**
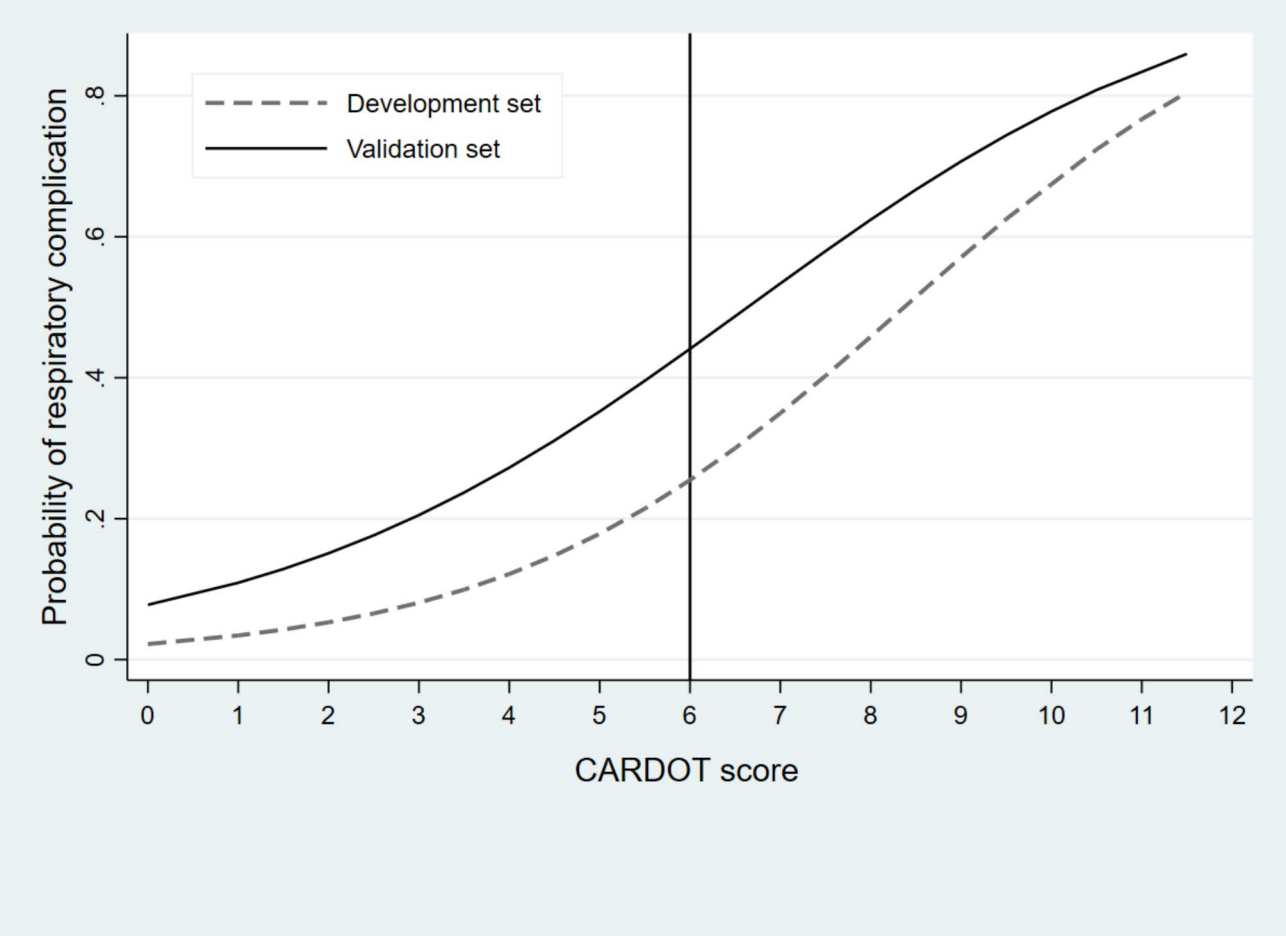
Score-predicted probability of respiratory complications using CARDOT score. The dash line and solid represented the risk of respiratory complications in the development and validation datasets. The probability of respiratory complications at the cutoff point of 6 (high risk group) in the validation dataset (45%)

**Fig. 5:**
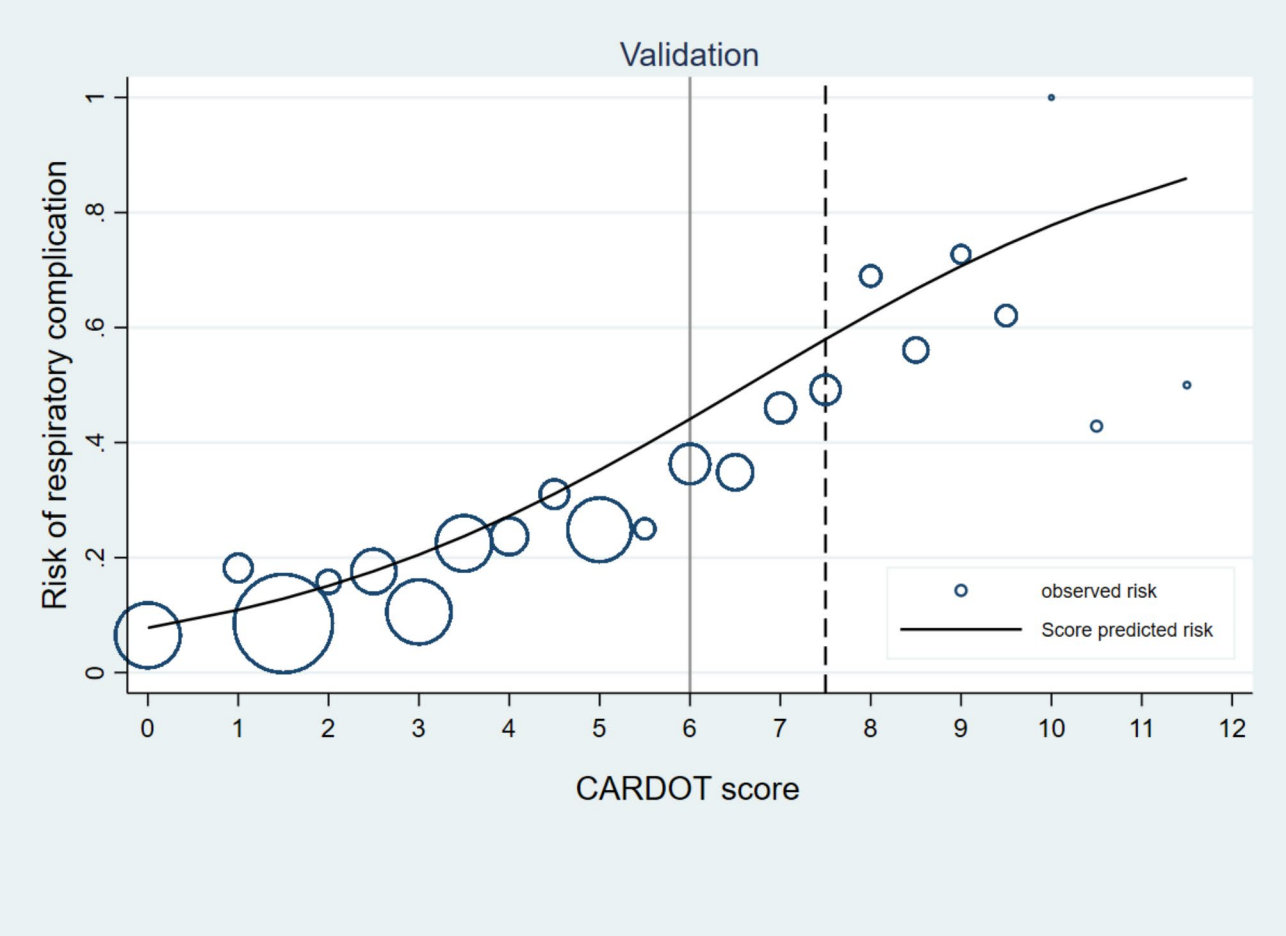
Calibration plots of the CARDOT score in the validation dataset. The dash line and solid represented the original threshold and adapted thresholds which categorized thoracic patients into low and high-risk groups of respiratory complications

**Fig. 6:**
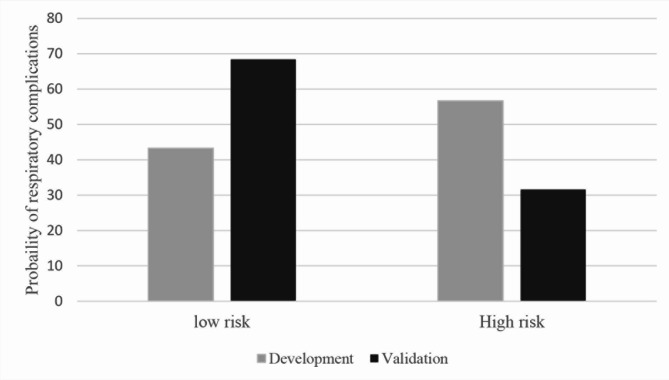
Probability of respiratory complication stratified by CARDOT score in the development and validation datasets

Patients with respiratory complications had a significantly greater proportion of COPD, lower preoperative SpO2, right-sided operation, thoracotomy, and longer duration of surgery than did those without respiratory complications. The odds ratio (OR) plots of the six CARDOT predictors in the development and validation datasets are presented in Fig. 2. The OR plots of the five parameters in both datasets showed similar results. The difference was not statistically significant in the right-sided operation (OR = 1.17, 95% CI 0.89–1.53) in the validation cohort. The most potent predictor of respiratory complications was low preoperative SpO2 (≤ 90%) (OR = 5.40, 95% CI 2.76–10.57). In the validation cohort, patients with respiratory complications had significantly greater NLRs than did those without respiratory complications (4.87 (2.49–8.26) vs. 2.55 (1.77–4.25), *P* < 0.001). The proportion of thoracic patients with a preoperative NLR ≥ 4.5 was 30% (494 of 1645 patients). The ROC curve of respiratory complications predicted by the risk scoring scheme of CARDOT was generated and shown in Fig. 3. A NLR ≥ 4.5 (high NLR) increased the risk of respiratory complications 3.68 times (OR = 3.68, 95% CI 2.89–4.69, *P* < 0.001) in the validation cohort. The AuROC curve of the validation cohort increased to 0.775 (95% CI 0.750–0.800) when combined with a high NLR. There was a statistically significant difference between the AuROC of patients in the validation dataset with and without a high NLR (*P* = 0.008). To improve the performance of the CARDOT score in identifying high risk patients, we adjusted the cutoff point for high-risk patients from 7.5 (original threshold) to 6 (adapted threshold). The distributions of patients with and without respiratory complications across risk categories (original vs. adapted thresholds) in both the development and validation datasets are shown in Supplementary Table S2 and Table 3, respectively. Figure 4 presents risk curves depicting the predicted probability of respiratory complications for the development and validation datasets using the adapted threshold. The performance measures for the original and adapted thresholds in the validation set were summarized in Table 4. The cutoff point was adjusted to ≥ 6 for high risk group, leading to a substantial increase to 31.60% while modestly decreasing specificity from 98.66 to 92.50% in the validation dataset. Additional details on model performance for various cutoff points are available in Supplementary Table S3. Figure 5 compares the calibration plots for the CARDOT score in the validation set using both the original and adapted thresholds. The plots show good agreement, with the observed events (represented by circles) closely following the diagonal line, particularly in the low-risk group. The Hosmer–Lemeshow goodness-of-fit statistics of the six predictors in the development and validation sets were significantly difference (*P* = 0.435 and 0.928, respectively). Probability of respiratory complications stratified by the CARDOT risk score in the development and validation datasets (Fig. 6). Proportion of respiratory events in development and validation datasets is shown in Table 5.

**Table 3:** Comparison of total CARDOT score and risk stratification between the development and validation datasets

Parameters	Development dataset (*n* = 1086)	Validation dataset (*n* = 1645)	*P*
Total scores (points)	2.2 (1.5–4.5)	3 (1.5-5.0)	< 0.001
**Original threshold**, ***n*(%)**			< 0.001
Low risk (≤ 7.5)	1005 (92.5)	1,593 (95.0)	
High risk (> 7.5)	81 (7.5)	52 (5.0)	
**Adapted threshold**, ***n*(%)**			< 0.001
Low risk (< 6)	839 (77.3)	1,433 (85.5)	
High risk (≥ 6)	246 (22.7)	212 (14.5)	

Data are presented as the median and 25^th^ and 75^th^ percentiles and the number of patients (%)

**Table 4:** Comparison of predictive performance for respiratory complications using original and adapted thresholds in validation dataset

Low vs. High risk group	Original threshold (≤ 7.5 vs. > 7.5)	Adapted threshold (< 6 vs. ≥ 6)
Sensitivity	9.46% (95% CI 6.68–12.91)	31.60% (95% CI 26.90–36.60)
Specificity	98.66% (95% CI 97.87–99.22)	92.50% (95% CI 90.90–93.90)
PPV	67.27% (95% CI 53.80-78.38)	55.20% (95% CI 48.20–62.00)
NPV	78.96% (95% CI 78.40–79.50)	82.30% (95% CI 80.20–84.30)
Positive likelihood ratio	7.08 (95% CI 4.01–12.49)	4.23 (95% CI 3.32–5.41)
Negative likelihood ratio	0.92 (95% CI 0.89–0.95)	0.74 (95% CI 0.69–0.79)

*PPV* positive prediction value, *NPV* negative positive value, *CI* confidence interval

**Table 5:** Proportion of respiratory events in development and validation datasets

Outcomes	Development dataset (*n* = 171)	Validation dataset (*n* = 370)	*P*
Postoperative bronchospasm	99 (9.1)	264 (16.1)	< 0.001
Respiratory failure	68 (6.3)	136 (8.3)	0.051
Desaturation	50 (4.6)	95 (5.8)	0.178
Atelectasis	33 (3.0)	51 (3.1)	0.926
Upper airway obstruction	8 (0.7)	27 (1.6)	0.039
Pneumonia	30 (2.8)	71 (4.3)	0.035
Adult respiratory distress syndrome	17 (1.6)	17 (1.0)	0.221

## Discussion

This study has externally validated the CARDOT score to predict respiratory complications after thoracic surgery. The CARDOT score is a risk assessment tool based on six factors: COPD, ASA PS, surgical side (right vs. left), surgery duration, preoperative SpO2, and thoracotomy procedure. The CARDOT score demonstrated acceptable discriminative performance in the validation dataset with an adapted CARDOT score. Incorporating a high NLR (> 4.5) significantly increased the predictive ability of CARDOT score. The incidence of respiratory complications in validation dataset had significantly higher than that of development dataset (22.5% vs. 15.8%), which is consistent with previous studies reporting complication rates between 7% and 25% [4, 5, 23, 25].

Several clinical predictors of respiratory complications after thoracic surgery have been reported, including advanced age [4–6, 9], current smoking [5, 25], COPD [6, 10, 14, 25], congestive heart failure [10], preoperative chemotherapy [2], low preoperative SpO2 [4, 6], low ppoFEV1 [4, 5], low ppoDLCO [2], prolonged surgery [9, 14, 25], and extensive pulmonary resection [4]. The present study supported that a high NLR increased the risk of respiratory complications approximately 3.7-fold. The threshold values for a high NLR differ depending on the specific condition or disease [16]. A previous study showed a significant association between elevated preoperative NLR (≥ 2.5) and poorer 5-year survival rates in patients undergoing resection for non-small cell lung cancer, with patients in the high NLR group having a significantly lower survival rate (47% vs 67.8%, respectively, *P* < 0.001) [20]. A retrospective study revealed that a high NLR (cutoff value of 2.8) was considered as a systemic inflammatory marker in an interstitial lung disease [26]. Our study demonstrated that a cutoff value of 4.5 yielded the best performance for discriminating patients with and without postoperative respiratory complications. This finding was consistent with those of previous studies varying between 2 and 5 [20, 26]. Further studies are recommended to determine the relationships between a high NLR and the incidence, severity and clinical outcomes of respiratory complications following thoracic surgery.

The development and validation cohorts exhibited significant differences in patient demographics and surgical characteristics. The validation dataset had a significantly lower incidence of COPD, fewer thoracotomies, and shorter duration of surgeries compared to the development dataset. The majority of surgical approach in validation cohort were performed via VATS (76.5%). These baseline discrepancies may have contributed to the CARDOT scoreʼs reduced performance in the validation dataset, as evidenced by lower AuROC (0.758 vs. 0.789) and PPV (67.3% vs. 91%). However, the positive likelihood ratio remained relatively stable (7.28 vs. 8.21), indicating consistent discriminative ability. To enhance clinical utility and focus on high risk patients, the CARDOT score was dichotomized using a cutoff of ≥ 6. This adaptation increased sensitivity to 31.60% (from 9.46%) while modestly reducing specificity to 92.50% (from 98.66%) compared to the original CARDOT score. Consequently, high risk patient identification rose from 5 to 14.5% in the validation dataset. Although false positives increased, the NPV improved from 78.9 to 82.3%, and the positive likelihood ratio remained acceptable (4.23). The predictive role of NLR was assessed solely in the validation cohort. Incorporating a high NLR significantly enhanced the discriminative performance of CARDOT score. These findings suggest that the adapted CARDOT score have some potential clinical implications. The increased sensitivity can lead to higher identification of patients at risk for respiratory complications, facilitating an early intervention and potentially improving clinical outcomes.

Although several pulmonary scoring systems for various surgical settings have been developed, the validated risk scores specifically designed for thoracic surgery are still limited. In 2010, Amar et al. reported two significant predictors of pulmonary complications, ppoDLCO and preoperative chemotherapy in CPRPC score following lung cancer resection [2]. Their study demonstrated that the accuracy of CPRPC was 0.630 in the development dataset and 0.470 in the external validation dataset [5]. Due to the low performance of CPRPCs, a new predictive risk score was modified by Yepes Termino et al. based on age, smoking status and ppoFEV1, with an AuROC of 0.740 (95% CI: 0.700–0.780) [5]. Recently, Zorrilla-Vaca et al. conducted an external validation study comparing the predictive performance of four risk scores—ARISCAT, LAS VEGAS, SPORC, and CARDOT—for postoperative respiratory complications in 2104 lung resection patients [27]. While the CARDOT score was specifically developed for thoracic surgery, the remaining three scores were primarily designed for other surgical contexts, with limited application in thoracic surgery (1.5-2%). Their primary outcomes were a composite of PPCs (atelectasis, pneumonia, respiratory failure, reintubation, discharge on supplemental oxygen (failure to wean), pulmonary effusion, pulmonary edema, and ARDS). All four risk scores demonstrated modest discriminative ability for PPCs, with AuROC values ranging from 0.60 (ARISCAT) to 0.68 (LAS VEGAS). Adding ppoFEV1 slightly improved the discriminative performance of LAS VEGAS (AuROC 0.70) and CARDOT (AuROC 0.68). Our study population differed significantly from the study of Zorrilla-Vaca et al., with younger patients (58 [47–66] years) vs. 67 [59–73] years), lower ASA PS 3 and 4 (93.1% vs. 24.8%), less proportion of COPD (33.3% vs. 11.2%), and a higher proportion of thoracotomies (23.5% vs. 6.7%). Additionally, our definition of PPCs excluded reintubation, oxygen dependency, and pleural effusion. These disparities may limit direct comparisons between our findings and those of Zorrilla-Vaca et al.

The CARDOT score offers several advantages for perioperative risk stratification. Primarily, it enables individualized risk assessment, allowing the anesthesiologist to discuss the likelihood of respiratory complications with patients during preoperative consultations. Secondly, preoperative optimization is crucial for COPD patients undergoing surgery. This includes bronchodilator therapy, corticosteroid administration and pulmonary rehabilitation. Also, thoracic patients presenting with preoperative hypoxemia (SpO2 < 96%) require careful evaluation and management to identify and address the underlying causes. Several studies reported that some surgical-specific risk factors including right-sided thoracic surgical procedures, prolonged duration of surgery and open thoracotomy, increased the risk of respiratory complications following thoracic surgery [9, 13, 14, 25]. For example, the occurrence of pulmonary edema after right pneumonectomy is caused by an impairment of lymphatic drainage, increased pulmonary blood flow to the left lung, disruption of endothelial cells of alveolar capillaries, and increased pulmonary permeability [28]. Therefore, several preventive strategies should be helpful for right-sided thoracic surgical procedures, including the minimization of operation time, the use of minimally invasive surgery, the use of one-lung ventilation with protective lung strategy (low tidal volume, titration of an optimal positive end-expiratory pressure (PEEP) and a lung recruitment maneuver) particularly in high-risk patients [29, 30]. Other preventive interventions for minimize PPCs include the use of quantitative neuromuscular monitoring, optimizing intraoperative fluid administration with goal-directed hemodynamic management and providing postoperative analgesia with various techniques of regional anesthesia if indicated [30]. Finally, CARDOT score can aid in medical resource allocation by identification of thoracic patients who are at risk for respiratory complications. Proactive allocation of high-dependency or intensive care unit resources should be considered, coupled with the use of invasive hemodynamic monitoring may be beneficial for enhanced perioperative surveillance and improvement of clinical outcomes in these patients [31]. High-dependency wards or intensive care units should be provided in advance for postoperative ventilatory support and the use of invasive hemodynamic monitoring should be considered to increase the level of perioperative surveillance in patients who are at risk of respiratory complications.

The main strength of this study was the use of an adequate sample size for the validation dataset. Our study also presented various aspects of model performance other than the AuROC curve. However, there were a few limitations in this study. This study was retrospective design and potential heterogeneity in respiratory complication definitions may have influenced the incidence of complications and an accuracy of the CARDOT score. To improve data quality issues, systematic data collection and strict inclusion/exclusion criteria were implemented. The absence of routine preoperative pulmonary function testing in our cohort (5% and 19.2% in development and validation datasets, respectively) limited our ability to assess its impact on the CARDOT score. Further research with larger sample sizes and comprehensive PFT data is warranted. Finally, the primary purpose of the CARDOT score is to identify patients at high risk of postoperative respiratory complications, enabling targeted interventions and enhanced perioperative monitoring. While the sensitive of this score was relatively low, its focus on specificity allows for efficient allocation of resources and prioritization of individual high risk patients.

## Conclusions

The CARDOT score demonstrated robust predictive performance in this temporal validation cohort, suggesting its continued utility in the era of minimally invasive thoracic surgery. As a readily available tool, this score can effectively identify patients at high risk of postoperative respiratory complications, particularly in settings with limited access to preoperative pulmonary function testing. While this study demonstrated the additive predictive value of a high preoperative NLR (≥ 4.5), further research with larger sample sizes is required to establish its role in risk stratification for thoracic patients.

### Electronic supplementary material

Below is the link to the electronic supplementary material.

Supplementary Material 1

Supplementary Material 2

Supplementary Material 3

## Data Availability

No datasets were generated or analysed during the current study.
