# Supplementary material for: External validation of the CARDOT score for predicting respiratory complications after thoracic surgery
Source: BMC Anesthesiol. 2024 Aug 30;24:301. doi: 10.1186/s12871-024-02685-5 (PMC11363378; doi:10.1186/s12871-024-02685-5)
Supplement: Supplementary file 2 — Supplementary Material 2 [file 12871_2024_2685_MOESM2_ESM.doc]

**Table S2**. Risk stratification in validation dataset (n=1645) by respiratory complications and total scores

| **Parameters** | **Respiratory complications (n = 370)** | **No complications (n = 1275)** | **OR (95% CI)** |
| --- | --- | --- | --- |
| Total CARDOT score | 4.5 (2.5-6) | 1.5 (1.5-3.5) | < 0.001 |
| **Threshold categories** |  |  |  |
| **Original threshold** |  |  |  |
| Low (≤ 7.5) | 122 (71.4) | 882 (96.5) | 1 |
| High (>7.5) | 49 (28.6) | 32 (3.5) | 11.07 (6.82,17.96) |
| **Adapted threshold** |  |  |  |
| Low (< 6) | 74 (43.3) | 765 (83.7) | 1 |
| High (≥6) | 97 (56.7) | 149 (16.3) | 8.56 (5.62,13.02) |

*OR* odds ratio, *CI* confidence interval
