# Supplementary material for: External validation of the CARDOT score for predicting respiratory complications after thoracic surgery
Source: BMC Anesthesiol. 2024 Aug 30;24:301. doi: 10.1186/s12871-024-02685-5 (PMC11363378; doi:10.1186/s12871-024-02685-5)
Supplement: Supplementary file 3 — Supplementary Material 3 [file 12871_2024_2685_MOESM3_ESM.doc]

**Table S3. **Performance of the CARDOT score threshold** **for predicting of respiratory complications****

| **Cut-off point** | **Sensitivity (%)** | **Specificity (%)** | **LR+** | **LR-** |
| --- | --- | --- | --- | --- |
| 1 | 96.31 | 13.22 | 1.11 | 0.28 |
| 1.5 | 94.46 | 15.28 | 1.11 | 0.36 |
| 2 | 83.21 | 45.15 | 1.52 | 0.37 |
| 2.5 | 82.10 | 46.61 | 1.54 | 0.38 |
| 3 | 77.49 | 51.97 | 1.61 | 0.43 |
| 3.5 | 71.59 | 64.36 | 2.01 | 0.44 |
| 4 | 62.18 | 72.42 | 2.25 | 0.52 |
| 4.5 | 57.93 | 75.80 | 2.39 | 0.56 |
| 5 | 54.61 | 77.63 | 2.44 | 0.58 |
| 5.5 | 40.96 | 87.88 | 3.38 | 0.67 |
| 6 | 39.67 | 88.84 | 3.55 | 0.68 |
| 6.5 | 32.10 | 92.13 | 4.08 | 0.74 |
| 7 | 26.38 | 94.78 | 5.06 | 0.78 |
| 7.5 | 21.03 | 96.34 | 5.75 | 0.82 |
| 8 | 15.50 | 97.76 | 6.91 | 0.86 |
| 8.5 | 11.01 | 98.17 | 6.45 | 0.90 |
| 9 | 7.56 | 98.99 | 7.52 | 0.93 |
| 9.5 | 4.61 | 99.27 | 6.30 | 0.96 |
| 10 | 1.29 | 99.77 | 5.65 | 0.99 |
| 10.5 | 1.11 | 99.77 | 6.30 | 0.96 |
| 11 | 0.55 | 99.55 | 12.10 | 0.99 |
| 11.5 | 0.18 | 99.95 | 4.03 | 0.99 |

*LR+* positive likelihood ratio, *LR-* negative likelihood ratio
